# Supplementary material for: Transcriptome Sequencing and Differential Analysis of Ovaries Across Diverse States (Follicular and Non-Follicular Phases)
Source: Animals (Basel). 2025 Aug 20;15(16):2436. doi: 10.3390/ani15162436 (PMC12382818; doi:10.3390/ani15162436)
Supplement: Supplementary file 1 [file animals-15-02436-s001.zip › Material S1.pdf]

S1 Real-time fluorescence quantification primers

Gene name	Primer name	Primer sequence (5 '-3')	Product size(bp)
GAPDH	GAPDH-F	GTCACCAGGGCTGCTTTT	208
	GAPDH-R	ATTTGATGTTGGCGGGAT	208
Actin	Actin-F	GAGGGAAATCGTGCGTGAC	145
	Actin-R	CTCGTTGCCGATGGTGAT	145
ENSECAG00000000548	ENSECAG00000000548-F	GCAGAGCACGAGAAGACAC	247
	ENSECAG00000000548-R	CAGGACCGACGGGAAGTT	247
COL1A1	COL1A1-F	AGGGCGACAGAGGCATTA	175
	COL1A1-R	CCTGGGAGACCGTTGAGT	175
EGR1	EGR1-F	AGTTTGCCAGGAGCGATGA	173
	EGR1-R	TGGGGATGGATAAGAGGTAGTA	173
TFPI2	TFPI2-F	GTCACAGCAATGAGAACCAG	187
	TFPI2-R	TTCCCTCCACAGCCAGTA	187
COL1A2	COL1A2-F	GCTTCAAGGGCATTAGGG	195
	COL1A2-R	CACTTCCATCGCTTCCAC	195
CCN1	CCN1-F	AGGAATGGGTCTGTGATGAG	239
	CCN1-R	GGACCACGAAGTTGTTTGA	239
COL3A1	COL3A1-F	GCCTCCCAGAACATCACG	83
	COL3A1-R	TTCAGGGCTTTCTTCACATT	83
HTRA1	HTRA1-F	AGTGGCTCTGGGTTTATCG	191
	HTRA1-R	AGCAGGACAGGCAGGTTT	191
SPARC	SPARC-F	CCTGCCAGAACCACCACT	119
	SPARC-R	CTTCTCAAACTCGCCAATG	119
FOS	FOS-F	CCCAACGGTAACTGCCATC	207
	FOS-R	TCCACCTTGCCCCTTCTGC	207
THBS4	THBS4-F	CTCCGCTACCTGAAGAATGA	179
	THBS4-R	GCTCTGGGGAGATTGTGAA	179
