# Supplementary material for: Transcriptome Sequencing and Differential Analysis of Ovaries Across Diverse States (Follicular and Non-Follicular Phases)
Source: Animals (Basel). 2025 Aug 20;15(16):2436. doi: 10.3390/ani15162436 (PMC12382818; doi:10.3390/ani15162436)
Supplement: Supplementary file 1 [file animals-15-02436-s001.zip › Material S2.pdf]

Supplementary Material 2

GO function bar top 20	Top 20 KEGG signaling pathways
cellular process（BP）	Neuroactive ligand-receptor interaction
single-organism process（BP）	Protein digestion and absorption
biological process（BP）	ECM-receptor interaction
regulation of biological process（BP）	Ovarian steroidogenesis
metabolic process（BP）	Parathyroid hormone synthesis, secretion and action
response to stimulus（BP）	cAMP signaling pathway
multicellular organismal process（BP）	Staphylococcus aureus infection
developmental process（BP）	Regulation of lipolysis in adipocytes
positive regulation of biological process（BP）	Phagosome
signling（BP）	P13K-Akt signaling pathway
localization（BP）	Dilated cardiomyopathy
cellular component organization or biogenesis（BP）	IL-17 signaling pathway
negative regulation of biological process（BP）	Amoebiasis
cell（CC）	Cocaine addiction
cell part（CC）	Arachidonic acid metabolism
organelle（CC）	Calcium signaling pathway
membrane（CC）	Pancreatic addiction
Membrane part（CC）	Cortisol synthesis and secretion
Organelle part（CC）	Mucin type O-glycan biosynthesis
binding（MF）	Aldosterone synthesis and secretion
